# Optimizing In Vitro Fertilization (IVF) Success in Hypergonadotropic Hypogonadism: A Case Study on the Impact of the Shanghai Protocol

**DOI:** 10.7759/cureus.54529

**Published:** 2024-02-20

**Authors:** Rokaiya Shaikh, Akash More, Shilpa Dutta, Namrata Choudhary, Jarul Shrivastava, Al Hera Ansari, Gauri Gajabe

**Affiliations:** 1 Clinical Embryology, Datta Meghe Institute of Higher Education and Research, Wardha, IND

**Keywords:** follicle-stimulating hormone, anti-mullerian hormone, abortion, infertility, pituitary gland

## Abstract

This article evaluated the effect of the Shanghai protocol on a hypergonadotropic hypogonadism patient undergoing in vitro fertilization (IVF) treatment. Hypergonadotropic hypogonadism was characterized by low sex hormone levels and elevated gonadotropins, leading to infertility. Poor ovarian response and failed pregnancy outcomes were the results of previous IVF treatments using conventional stimulation methods. The 37-year-old female patient was advised to follow the Shanghai protocol, which involved gonadotropin stimulation following pituitary suppression with a long-acting gonadotropin-releasing hormone agonist (GnRH-a). The Shanghai protocol significantly improved the ovarian response. Two oocytes were retrieved, and one 4AA grade (number 4 represents an expanded blastocyst, the embryo is large, and the zona is thin; first A represents the inner cell mass of numerous and tightly packed cells; second A represents trophectoderm, with many cells organized in epithelium) embryo was formed. According to her previous result, the patient with hypergonadotropic hypogonadism who had one unsuccessful IVF cycle after visiting our infertility center was advised of the Shanghai protocol. Establishing these results and enhancing the Shanghai protocol's implementation to this specific patient treatment, clinical pregnancy was achieved.

## Introduction

Infertility is the failure to achieve a pregnancy after 12 months or more of regular unprotected sexual intercourse. Global estimates place the proportion of reproductive-age couples at 8-12%. Worldwide, secondary infertility comprises the majority of occurrences and is usually triggered by illnesses of the reproductive system [1]. The Shanghai protocol is a complete therapy program designed particularly for hypergonadotropic hypogonadism patients that involves several elements such as ovarian stimulation, oocyte retrieval, transfer of embryos, and luteal stage support. This protocol aims to optimize the ovarian response, increase oocyte quality and quantity, improve fertilization rates, and enhance embryo implantation potential, ultimately maximizing the chances of a successful pregnancy for these individuals [2].

Through a complex process, the pituitary gland regulates the secretion of follicle-stimulating and luteinizing hormones. According to the available data, luteinizing hormone-releasing hormone (LH-RH) contributes to the synthesis and storage of both follicle-stimulating and luteinizing hormones. Furthermore, it is a well-known fact that testosterone and estradiol may hinder the release of these hormones [3].

The abnormal secretion of gonadotrophins from the pituitary can cause gonadal failure and lead to hypergonadotropic hypogonadism, which can cause infertility. Hypergonadotropic hypogonadism may result from either absent or inadequate hypothalamic gonadotropin-releasing hormone (GnRH) secretion or failure of pituitary gonadotropin secretion. This condition has been related to several congenital and acquired causes, including functional and organic forms [4].

In this paper, we delved into the effect of the Shanghai protocol on hypergonadotropic hypogonadism patients undergoing in vitro fertilization (IVF) treatment. This case study focuses on a patient who had hypergonadotropic hypogonadism and who managed to conceive via IVF treatment confirmed by the clinical pregnancy test.

## Case presentation

Medical history

The couple included a 37-year-old female and her 39-year-old male partner in this case study. For the past two years, the couple has been unable to conceive after regular unprotected sexual intercourse. The female patient had a history of hypergonadotropic hypogonadism, a disorder distinguished by low sex hormone levels and elevated gonadotropin levels, as shown in Table 1. The clinical symptoms, hormonal profile, and imaging tests were used to establish the diagnosis.

**Table 1:** Female's hormonal values

Female hormonal parameters	Values
Estrogen	15 pg/mL
Follicle-stimulating hormone (FSH)	2.5 mUI/mL
Luteinizing hormone (LH)	1.3 IU/L
Anti-Müllerian hormone (AMH)	0.02 ng/ml
Antral follicular count (AFC)	2

The female patient faced difficulty in achieving pregnancy due to irregular menstrual cycles. Ultrasonography revealed small ovaries, i.e., 1.8 cm in length, 1 cm in height, and 0.8 cm in width (the normal size of the ovary is 3 cm in length, 2.5 cm in height, and 1.5 cm in width) with a reduced number of antral follicles. The male partner had also undergone a thorough evaluation, including a semen analysis, which showed normal sperm parameters, as shown in Table 2.

**Table 2:** Semen parameters

Semen parameters	Values
Count	60 mil/ml
Motility	65%
Defect	80%
Normal morphology	20%

The couple then underwent one attempt at IVF using a conventional stimulation protocol. However, the attempt resulted in a low number of oocytes retrieved, and one oocyte of poor quality can cause unsuccessful pregnancy outcomes due to poor ovarian response.

We reviewed the patient's medical history at our infertility center, and it was found that she was G0P0L0A0 (where G represents gravida, P represents preterm, L represents a living child, and A represents abortion), indicating that she had three previous pregnancies, with one reaching viability. However, she went through one abortion procedure and two sporadic abortions.

The patient had an issue with the pituitary gland, which secretes follicle-stimulating hormones and luteinizing hormones. The levels of both hormones were very high. The patient's luteinizing hormone value was 104 mlU/mL, and the follicle-stimulating hormone value was 107 mlU/ml. The patient's anti-Müllerian hormone (AMH) value was 0.01 ng/mL. This indicated that the patient's antral follicle count and level of AMH had been determined to be extremely poor. The diminished ovarian reserve was confirmed by a lower AMH level, which means fewer oocytes were available for fertilization [5]. However, double stimulation was used for the follicles in the Shanghai procedure [6]. In conventional protocol, the doctor gave medication to grow follicles in the fluid. Then, ovum pick-up was done, and one germinal vesicle (GV) poor-quality oocyte was retrieved from the ovaries. Intracytoplasmic sperm injection (ICSI) was not performed on the GV oocyte.

Given the history of poor ovarian response in previous IVF cycles, the decision was made to switch to the Shanghai protocol, a novel ovarian stimulation protocol. The Shanghai protocol involved the administration of a long-acting gonadotropin-releasing hormone agonist (GnRH-a) for pituitary suppression, followed by gonadotropin stimulation.

Clinical findings

The patient diagnosed with hypergonadotropic hypogonadism presented for IVF treatment. Prior IVF treatments that used conventional stimulation protocols had poor ovarian responses and failed pregnancies. The Shanghai protocol, a novel ovarian stimulation protocol, was chosen as a replacement due to the patient's medical history.

Under the Shanghai protocol, the patient received a long-acting GnRH-a for pituitary suppression, followed by gonadotropin stimulation. Throughout treatment, the ovarian response was carefully observed. Transvaginal ultrasound scans demonstrated a notable improvement in follicular development, with numerous mature follicles being seen [7]. A positive ovarian response to the Shanghai protocol was indicated by the serum hormone levels, which showed a strong endogenous gonadotropin response.

Generally, we administered single stimulation of gonadotropins, but in the Shanghai protocol, we utilized double stimulation for the follicles. The luteal phase and the early follicular phase are the two phases. On day two, the patient was treated with letrozole and clomiphene citrate. After an interval of four days, the patient was treated with human menopausal gonadotropin (HMG). After medication and stimulation, the follicles were observed. On day seven, the patient was treated with a GnRH-a, marking the second stimulation. After completing stimulation, we evaluated the follicles using ultrasonography and observed that the follicle size was 18 mm. The patient was again treated with gonadotropin-releasing hormone. After 36 hours, the patient underwent ovum pickup [8].

In previous cycles, one oocyte of very poor quality was found during the oocyte retrieval procedure. Compared to previous oocytes, two oocytes were retrieved in total. The retrieved oocytes were fertilized using ICSI with the partner's sperm, resulting in successful fertilization and the production of embryos of 4AA grade.

One 4AA (number 4 represents the expanded blastocyst; the embryo is large and the zona is thin; first A represents the inner cell mass of numerous and tightly packed cells; second A represents trophectoderm, with many cells organized in epithelium) embryo was successfully transferred into the patient's uterus on day five of embryo development. Progesterone supplementation was provided to support endometrial receptivity and implantation.

**Figure 1:**
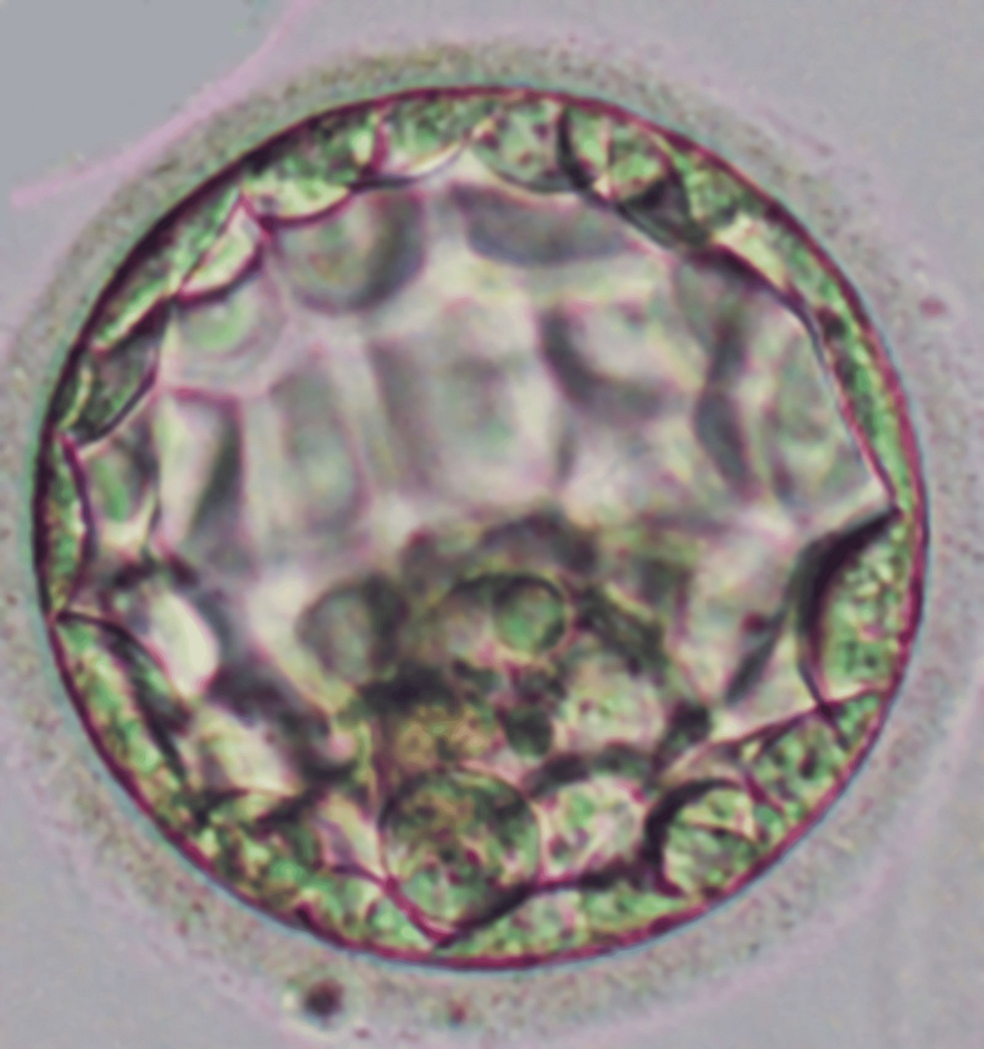
4AA embryo transferred to the patient.

Throughout the treatment, the female patient and her male partner were closely monitored for any potential adverse effects or complications. Regular communication and counseling were provided to address any concerns and ensure compliance with the treatment plan. No complications were encountered during the procedure, as shown in Figure 1.

Follow-up

After 14 days of embryo transfer, the patient came for the follow-up. During follow-up, blood samples in the EDTA tube were sent to the biochemistry lab, and the report results were beta-human chorionic gonadotropin (β-hCG) positive, i.e., 1050 mUI/mL. The doctor then advised the patient to eat healthy food, think positively, live stress-free, and about patient self-care.

## Discussion

First proposed by Kuang et al., mild stimulation at the follicular phase was followed by ovarian stimulation at the luteal phase to maximize oocyte retrieval. Their initial investigation on 38 poor ovarian response (POR)-afflicted women revealed that the average quantity of follicles, retrieved oocytes, and metaphase II oocytes acquired during the Shanghai protocol were in the order of 3.9, 2.6, and 2.05, respectively, which was reasonably positive [8]. In this regard, our result found only a single patient, and the result came out positive.

The study conducted by Liu et al. on women with POR who were over 38 years old revealed that the number of retrieved oocytes and live embryos was twice as many as during the ovarian stimulation follicular phase. These results indicate that through the Shanghai protocol, a positive outcome was achieved, which involved dual gonadotropin stimulation phases, utilizing letrozole, clomiphene citrate, and human menopausal gonadotropin [7,9].

By the Bologna criteria, the poor responders were a subset of patients with a relatively poor prognosis for pregnancy, and their live birth rate in natural-cycle IVF was continuously low, ranging from 6.8% to 7.9% [8].

The monitoring was conducted using serum hormone levels and ultrasound scans. The number of retrieved oocytes increased significantly with ovum pick-up, with ICSI performed resulting in two excellent embryos. Also, progesterone supplementation added one embryo transfer successfully. Alongside, there was close monitoring, good communication, and counseling for both partners, all of which contributed to peaceful treatment plan compliance.

## Conclusions

This patient had hypergonadotropic hypogonadism undergoing IVF treatment and showed significant improvements in ovarian response, embryo quality, and pregnancy success when the Shanghai protocol was used. The Shanghai protocol's specific approach combining long-acting gonadotropin and GnRH-a stimulation was successful in increasing ovarian stimulation to improve IVF results. According to this research, patients with hypergonadotropic hypogonadism who had previously failed IVF cycles due to poor ovarian response may find the Shanghai protocol to be an effective option. The limitation of this case study examination was the inclusion of only one patient.
